# Arachidonic Acid Enhances Reproductive Performance and Oocyte Quality of Female Blotched Snakehead (*Channa maculata*) via Activation of the TGF-β/Smad Signaling Pathway

**DOI:** 10.3390/ijms27198540

**Published:** 2026-09-24

**Authors:** Shuzhan Fei, Jiahui He, Tao Yang, Junhao Zhang, Mi Ou, Haiyang Liu, Xincheng Zhang, Qing Luo, Jian Zhao

**Affiliations:** 1 Key Laboratory of Tropical and Subtropical Fishery Resources Application and Cultivation, Ministry of Agriculture and Rural Affairs, Pearl River Fisheries Research Institute, Chinese Academy of Fishery Sciences, Guangzhou 510380, China; feisz@prfri.ac.cn (S.F.);; 2 College of Fisheries and Life Science, Shanghai Ocean University, Shanghai 201306, China; 3 School of Life and Health Sciences, Hunan University of Science and Technology, Xiangtan 411201, China

**Keywords:** highly unsaturated fatty acids, *Channa maculata*, steroid hormone, TGF-β/Smad signaling pathway, egg quality, broodstock

## Abstract

Arachidonic acid (ARA) is an indispensable fatty acid for aquatic organisms, playing critical roles in reproductive physiology. This study aimed to evaluate the effects of graded dietary ARA levels on the reproductive performance, tissue fatty acid profiles, plasma sex hormone and prostaglandin concentrations, ovarian molecular responses, and offspring larval quality in female blotched snakehead (*Channa maculata*). Three experimental diets with ARA levels of 0% (control, C), 1.5% (ARA1.5), and 3.0% (ARA3.0) were formulated and administered to female blotched snakehead for 12 weeks. The results demonstrated that dietary ARA supplementation significantly improved fertilization rate, hatching rate, and larval growth performance, while reducing the larval malformation rate. ARA supplementation enhanced the deposition of ARA, eicosapentaenoic acid (EPA), and docosahexaenoic acid (DHA) in both ovaries and muscle tissues. Plasma levels of sex hormones and prostaglandins (PGE2, PGF2α) were significantly elevated with increasing dietary ARA concentrations. Furthermore, qRT-PCR and Western blot results indicated that ARA supplementation upregulated the expression of genes and proteins in the TGF-β/Smad signaling pathway and steroidogenesis-related genes. Metabolomic analysis identified differential metabolites enriched in arachidonic acid metabolism, the VEGF signaling pathway, and glycolysis/gluconeogenesis in ovaries and larvae, with ARA-derived eicosanoids significantly upregulated. Dietary ARA supplementation was associated with improved reproductive performance, accompanied by reproducible molecular alterations in the ovarian TGF-β/Smad signaling pathway, indicating that this cascade represents a plausible underlying regulatory mechanism for this improvement.

## 1. Introduction

The gonadal development of fish is fundamental to the efficiency of aquaculture production, as they are influenced by various factors including the genetic background of broodstock, nutrient availability, feeding strategies, and management practices. Of these, oocyte quality directly determines embryonic developmental potential and overall reproductive efficiency [1,2]. Currently, it is widely acknowledged that a rich composition of polyunsaturated fatty acids (PUFA), a high content of protein with appropriate amino acids, and certain bioactive factors (such as carotenoids, vitamins, and minerals) are essential for the gonadal development of fish [3,4,5]. Arachidonic acid (ARA) is an essential fatty acid for aquatic animals and a key component of cell membrane phospholipids [3,6]. The roles of ARA and its metabolites in regulating growth and reproduction, immunity, oxidative stress, lipid metabolism, and bone development have been preliminarily confirmed [7,8]. ARA serves as a crucial precursor for the synthesis of eicosanoids, particularly prostaglandin E2 (PGE2) of the series II. Prostaglandins and thromboxanes, collectively referred to as prostanoids, are bioactive lipid mediators derived from fatty acids that exert vital functions in various physiological processes [9]. They are synthesized via the enzymatic action of cyclooxygenases (COX) and associated enzymes on C20 polyunsaturated fatty acids released from membrane phospholipids [10].

Arachidonic acid (C20:4n-6) is an ω-6 highly unsaturated fatty acid (HUFA) that plays a critical physiological role in the sexual maturation of fish by activating nuclear transcription factor signaling pathways to mediate cellular signal transduction, regulate lipid metabolism and membrane lipid composition during gonadal development [11,12]. In recent years, the functional role of ARA in animal reproduction has attracted growing attention. The key regulatory pathways include the cyclic adenosine monophosphate (cAMP), protein kinase A (PKA), and protein kinase C (PKC) pathway, alongside bioactive lipid mediators generated from ARA metabolites (in particular PGE2) and steroid hormones, all of these factors are well-established as essential regulators of oocyte maturation [13]. For example, dietary supplementation with 10 g/kg ARA may promote gonadal development in femalerice field eel (*Monopterus albus*) broodstock by enhancing the PGE2 downstream signaling pathway, thereby regulating steroid hormone synthesis and vitellogenin (VTG) production [14]. Dietary supplementation with ARA (10.0 g/kg) significantly increased plasma estradiol (E2) levels and upregulated hepatic *vtg* gene expression in female Chinese sturgeon (*Acipenser sinensis*) [15]. In female yellow catfish (*Pelteobagrus fulvidraco*), dietary supplementation of ARA at 4.67% and 10.07% of total fatty acids enhanced sex hormone levels, spawning performance, and offspring quality [16]. Similarly, supplementing the diet of largemouth bass (*Micropterus nigricans*) broodstock with 3.6% ARA (in combination with total fatty acids) improved the growth performance and physiological health status of their offspring [17]. Thus, incorporating an adequate level of ARA into the diet is crucial for freshwater fish, particularly during key physiological phases, given its significant structural and functional contributions to gonadal development.

Previous studies have reported that both transforming growth factor beta 1 (TGF-β1) signaling and ARA metabolic pathways are significantly enriched in the ovary of the blotched snakehead, and *tgf-β1* is significantly upregulated in well-developed ovaries [18]. TGF-β1, a key member of the TGF-β superfamily, plays a significant role in cell differentiation, tissue morphogenesis, apoptosis, and embryonic development [19,20]. Additionally, TGF-β1 has been found to play a significant role in the maturation of primary growth oocytes into previtellogenic oocytes in zebrafish (*Danio rerio*) [21]. Therefore, we hypothesize that TGF-β1 plays a critical role in oocyte proliferation and development, and that suppression or interference of key genes in this pathway may lead to reproductive dysfunction. Accordingly, elucidating the molecular mechanism through which TGF-β signaling mediates ARA regulation of gamete quality and fertilization capacity is of great significance.

Snakehead is a commercially valuable freshwater fish species widely cultivated in China. In recent years, artificial propagation of blotched snakehead has encountered multiple bottlenecks, including delayed gonadal development and asynchronous oocyte maturation, leading to low fertilization rates and inferior fry quality [22]. Concurrently, the development of broodstock-specific formulated diets remains insufficient, which hampers large-scale, efficient production of fish larvae. Therefore, this study used blotched snakehead to investigate the mechanism by which ARA regulates ovarian development and oocyte maturation via the TGF-β signaling pathway, aiming to provide a theoretical basis for improving reproductive performance and egg quality of economically important fish species.

## 2. Results

### 2.1. Reproductive Performance and Larval Quality

As shown in Table 1, the HSI of females in the ARA3.0 group was notably higher than that of the control group (*p* < 0.05). However, dietary levels of ARA did not have a significant impact on the GSI or fecundity of broodstock. Eggs from the control group showed a significantly larger diameter than those in the groups supplemented with ARA (*p* < 0.05). In contrast, the supplementation of dietary ARA significantly enhanced the fertilization rate compared to the control group (*p* < 0.05). Compared with the control group, the hatching rates of the ARA1.5 and ARA3.0 groups were significantly higher (*p* < 0.05). The malformation rate in both the control and ARA1.5 groups was significantly higher than that in the ARA3.0 group (*p* < 0.05). During the endogenous trophic stage, the 3 DPH larvae in the ARA1.5 group exhibited significantly greater body length compared to those in the control group (*p* < 0.05). Furthermore, 7 days after hatching, both body length and body weight were significantly greater in the ARA-supplemented group compared to the control group (*p* < 0.05).

### 2.2. Fatty Acid Analysis of Ovaries and Muscle

To analyze the composition and content distribution of fatty acids in female tissues (ovary and muscle), the fatty acid profiles of these tissues are shown in Table 2. Dietary ARA levels did not significantly affect the saturated fatty acid (SFA) and monounsaturated fatty acid (MUFA) contents in the ovary and muscle among the three treatments. The concentrations of ovarian C18:2n-6 (linoleic acid) and C20:2n-6 (eicosadienoic acid) decreased with increasing dietary ARA concentration (*p* < 0.05). In contrast, the contents of C20:4n-6 (ARA), C20:5n-3 (eicosapentaenoic acid, EPA), and C22:6n-3 (docosahexaenoic acid, DHA) in the ovaries increased with rising dietary ARA levels (*p* < 0.05). The concentrations of various fatty acids in muscle were considerably lower than those in the ovaries. Dietary ARA supplementation significantly influenced PUFA profile in muscle tissue compared with the control group, with marked increases in ARA, EPA, and DHA observed as dietary ARA levels increased (*p* < 0.05).

### 2.3. Immunohistochemical Analysis

Immunohistochemical staining of ovarian tissue specimens revealed the expression level and spatial distribution of target proteins. Immunoreactivity for ER and CYP19A1 was observed in follicular cells and oocyte membranes, with no positive signal detected in negative control samples. As shown in Figure 1A, compared with the control group, both ER and CYP19A1 were predominantly localized to the oocyte membranes and surrounding follicle cells, showing markedly stronger immunostaining in the ARA3.0 group. As shown in Figure 1B,C, quantitative analysis demonstrated that the ovarian immunoreactive optical density of CYP19A1 and ER was significantly lower in the control group than in the ARA3.0 group (*p* < 0.05).

### 2.4. Plasma Levels of Sex Hormones and Prostaglandins

Plasma steroid hormone levels reflected the regulatory effect of dietary ARA on ovarian development in female blotched snakehead. As shown in Figure 2, dietary ARA contents significantly affected the serum steroid hormone and prostaglandin levels in female blotched snakehead among the three treatments. The ARA1.5 group showed markedly elevated concentrations of LH and E2 when compared with the control group (*p* < 0.05). PGF2α content was markedly elevated by dietary ARA supplementation (*p* < 0.05). Furthermore, a dose-dependent increase in 11-KT and PGE2 levels was observed with increasing dietary ARA intake (*p* < 0.05).

### 2.5. Ovarian Development-Related Gene Expression and TGF-β/Smad Signaling Pathway

Compared with the control group, dietary supplementation with 3.0% ARA significantly upregulated the relative expression of *tgfb1* and *tgfb3* in the ovary, and subsequently activated the expression of downstream regulatory factors *smad2* and *smad3* (*p* < 0.05) (Figure 3A). The expression of genes associated with steroid hormone synthesis was notably influenced by dietary ARA levels. Compared with the control and the ARA3.0 group, the expression of *cyp17a1* in the ARA1.5 group was significantly upregulated (*p* < 0.05). The relative expression levels of *cyp19a1*, *er*, *pr*, *foxo1*, and *foxo3* genes in the ARA3.0 group were significantly higher compared to those in the control and the ARA1.5 group (Figure 3B). Meanwhile, the ARA-supplemented group exhibited a significantly higher TGF-β1 protein level compared with the control group (*p* < 0.05). Dietary supplementation with ARA, especially at the 3% content, significantly elevated the protein expression levels of Smad2, Smad3, and Smad4 (*p* < 0.05) (Figure 3C,D).

### 2.6. Metabolic Profile and Cluster Analysis of Differential Metabolites

To further investigate the regulatory effect of dietary ARA on ovarian development, we performed a lipidomic analysis to characterize the classes, distribution and functional profiles of lipids. As illustrated in Figure S1, the OPLS-DA score plots reveal distinct separation between the control and ARA3.0 groups in both ovarian and 3DPH larvae. The model exhibited stable R2Y and Q2Y values, indicating good fitness and robust predictive capability, thereby supporting the reliability of subsequent differential metabolite analysis. As shown in Figure 4A,B, 278 metabolites were significantly increased, and 211 metabolites were significantly decreased in the ovaries between the C and ARA3.0 groups. In the 3DPH larvae, 401 metabolites were significantly upregulated, and 294 were significantly downregulated compared to the control group. The contents of norlinolenic acid, N-[2-(5-methoxy-3H-indol-3-yl)ethyl]acetamide, 2-ethyl-1-methylpyridin-4-one, vanillin acetate, and ethoxyquin in the ARA3.0 group were markedly enhanced in contrast to the C group, whereas the contents of PIP (20:0/PGF1), 7-ketocholesterol, dihydrolipoate, richter, and L,L-cyclo (leucylprolyl) were markedly reduced in ovarian tissue. In the 3DPH larvae, the contents of RCS-4 3-methoxy isomer, acetyl-arginyl-glycyl-aspartyl-serinamide, lyso PE(0:0/14:1), 12-trans-hydroxy juvenile hormone III, and 9,10-epoxyoctadecanoic acid in the ARA3.0 group were markedly enhanced, while PI(18:0/22:4), 5-methylcytosine, 5-methylthio-D-ribose, asperulosidic acid, eugenitol, and vetiveryl acetate were markedly reduced.

KEGG pathway enrichment analysis provides valuable insights, indicating that significantly different metabolites are potentially involved in metabolic pathways and biochemical synthesis processes (Figure 4C,D). In the ovary, ARA3.0 treatment significantly upregulated arachidonic acid metabolism, the pentose phosphate pathway, lipoic acid metabolism, retinol metabolism, VEGF signaling pathway, and apoptosis, while downregulating parathyroid hormone synthesis, secretion, biological activity, and the AMPK signaling pathway, compared with the control group. In the 3DPH larvae, differential metabolites were significantly enriched in pathways related to glycolysis/gluconeogenesis, ARA metabolism, lysine biosynthesis, and the regulation of the actin cytoskeleton, VEGF signaling pathway, insulin secretion, gastric acid secretion, and pancreatic secretion, while significantly downregulating pathways associated with cortisol synthesis and secretion, Cushing syndrome, apoptosis, and rheumatoid arthritis.

Hierarchical clustering analysis was performed on lipids and lipid-like differential metabolites significantly enriched in ovaries and larvae, respectively (Figure 4E,F). In the ovary, these differential metabolites were primarily involved in processes such as ARA metabolism, steroid hormone biosynthesis, and fatty acid biosynthesis. Compared with the control group, the relative abundances of taurocholic acid, taurodeoxycholic acid, PC(14:0/22:5), maleamic acid, furanodiene, phytocassane B, thromboxane B2, prostaglandin I2, prostaglandin F1, 12-keto-leukotriene B4, glutarylcarnitine, dehydroabietic acid, neuraminic acid, phosphatidylserine, prostaglandin J2, and eicosapentaenoic acid were significantly up-regulated in the ARA3.0 group. However, triglyceride metabolites such as glycerophosphatidylcholine, methyltaurocholate, lysophosphatidylcholine, monoglyceride, phosphatidylcholine, and phosphatidylserine were significantly down-regulated. In the 3DPH larvae, cluster heat map analysis showed that the differential metabolites were primarily associated with ARA metabolism, glycolysis/gluconeogenesis, and linoleic acid metabolism. Metabolites such as lyso-phosphatidylinositol (0:0/18:0), leukotriene-D4, lyso-phosphatidylcholine (18:2/0:0), isopimaric acid, 6-keto-prostaglandin E1, 6-keto-prostaglandin F1 alpha, prostaglandin 12, thromboxane B2, 12-keto-leukotriene B4, lyso-phosphatidylcholine (20:5/0:0), lyso-phosphatidylethanolamine (0:0/22:6), phosphoethanolamine, phosphatidylserine [16:0/18:2 (9Z,12Z)], phytocassane B, 3a,20b-pregnanediol, and prostaglandin J2 were significantly up-regulated in the ARA3.0 group. In contrast, metabolites such as copalic acid, gestrinone, 18-hydroxycorticosterone, phylloquinone, prednisone, 17alpha-hydroxypregnenolone, pregnanolone, monoglyceride (0:0/15:0/0:0), lyso-phosphatidylcholine (22:5/0:0), avenacin B2, misoprostol acid, phosphatidylinositol [16:0/22:2(13Z,16Z)], 1-monostearin, 7-ketocholesterol, and vetiveryl acetate were remarkably down-regulated compared with the control group.

## 3. Discussion

Broodstock cultivation essentially involves a continuous process of nutrient accumulation by the organism, which is crucial for ensuring gonadal development, fertilization, spawning, and larval quality [23]. However, insufficient or unbalanced nutrition may cause ovarian reabsorption, arrest ovarian development, and reduce reproductive activity [24]. In the past decade, increasing efforts have been dedicated to understanding the regulatory role of ARA in the reproductive processes of aquatic animals. It has been observed that during specific gonadal development stages, ARA is selectively retained and transported to the gonads [25,26]. In this study, a dietary ARA level of 3.0% (13.45% of total fatty acids) markedly enhanced both the fertilization rate and hatching rate of female blotched snakehead, and significantly reduced the malformation rate of larvae after hatching. Notably, dietary ARA supplementation significantly reduced egg diameter. Furthermore, continuous monitoring of larval body length and body weight at 3 days and 7 days post-hatching during the endogenous trophic phase confirmed that ARA supplementation significantly promoted the growth of offspring larvae. This improvement can be attributed to the accumulation of higher-quality or greater quantities of endogenous nutrients in the vitellus. In a previous study on the broodstock of northern snakehead (*Channa argus*), it was found that an adequate protein supply from the female led to sufficient accumulation of amino acids and fatty acids in the ovaries, enabling the larvae at 14 and 21 days post-hatching to exhibit better growth performance [27]. Numerous studies have demonstrated that dietary ARA requirements exhibit significant variation among fish species. Moreover, dietary ARA supplementation exerts a positive influence on multiple reproductive performance parameters, including egg size, fertilization rate, hatching rate, deformity rate, and larval quality [16,28,29]. Some studies have reported that the dietary ARA levels required for optimal reproductive performance were 0.6% for Japanese flounder (*Paralichthys olivaceus*) [30] and 1.6–2.1% for blue gourami (*Trichopodus trichopterus*) [31], respectively. Additionally, it has been demonstrated that a 3.0% ARA dietary level had a negative impact on egg quality in the virgin pikeperch (*Sander lucioperca*), a negative effect attributed to an imbalance in essential fatty acid requirements resulting from a change in the ARA/EPA ratio [32].

In this study, the contents of ARA, EPA, and DHA in muscle increased significantly as the dietary ARA level increased. In addition, we discovered that dietary ARA supplementation notably enhanced the deposition of LC-PUFAs in the ovary. The ARA content in the ovary rose as the dietary ARA level increased. Conversely, the content of linoleic acid, its biosynthetic precursor, exhibited an opposite trend. These observations imply that blotched snakehead may possess the capacity to synthesize ARA from linoleic acid via elongase–desaturase enzymatic systems, which needs further molecular validation. DHA and EPA play crucial roles in the development of eggs and larvae, whereas ARA serves as a precursor for eicosanoids that regulate reproductive processes and oocyte maturation [33]. DHA exists predominantly as phosphatidylcholine in fish eggs and gets incorporated into neural tissues and retinal membranes during larval development, fulfilling a crucial structural function in the developing organism [34]. Freshwater teleosts can biosynthesize LC-PUFAs from linoleic acid and α-linolenic acid via desaturation and elongation pathways. In contrast, marine teleosts are generally unable to perform this conversion, largely because they lack the Δ5-desaturase enzyme required for these metabolic steps [35,36]. However, several studies have demonstrated that, owing to the competitive substrate inhibition between EPA and ARA, when a substantial quantity of ARA accumulates in the body, the content of EPA will decline [24]. Therefore, it is recommended to supplement ARA during the sexual maturity stage of carnivorous snakeheads. Meanwhile, the balance between ARA and EPA should be considered to meet the reproductive requirements of these fish.

Gonadal steroidogenesis, a crucial aspect of reproductive physiology, has been thoroughly investigated, with a particular focus on ARA, which is recognized as a key regulator in this pathway. In the current study, dietary supplementation of ARA significantly increased the plasma levels of steroid hormones and prostaglandins in female blotched snakehead. Moreover, the levels of 11-KT and PGE2 increased in tandem with the rise in dietary ARA level. Previous studies on female yellow catfish have also demonstrated that dietary ARA (10.7% of total fatty acids) can increase plasma E2 and follicle-stimulating hormone (FSH) concentrations, as well as promote the development and maturation of ovarian follicles [16]. Both ARA and EPA contribute to reproductive functions via the biosynthesis of eicosanoids [5,37]. Consistent with our observation, a comparable elevation in PGE_2_ and 5-HETE levels coupled with elevated dietary ARA content has been documented in the liver of F_2_ generation Chinese sturgeons [15]. Analogous elevations have also been reported in the liver of juvenile grass carp [38]. In vitro and in vivo studies conducted on European sea bass have demonstrated that free ARA induces maturation and potentiates gonadotropin-induced maturation in a dose- and time-dependent manner. Moreover, PGs might play a crucial role in the induction of maturation and the courtship between male and female fish during spawning. Indeed, PGE2 has been found to be important for the induction of maturation [39]. It was found that the plasma concentrations of PGE3 and PGF3α decreased in parallel with increasing dietary ARA levels in Senegalese sole (*Solea senegalensis*) [40]. In mammals, it has been demonstrated that ARA supplementation seems to increase plasma steroid concentrations, either directly by enhancing the availability of steroidogenic substrates or indirectly through derivatives of PUFA, such as prostaglandins [41]. These studies suggest that sex steroid hormones are affected by the feedback modulation of ARA through its influence on the steroidogenesis pathway.

In this study, we carried out a more comprehensive and in-depth investigation into the impacts of dietary ARA levels on genes associated with the regulation of ovarian steroidogenesis, as well as the role of TGFβ/Smad signaling in the molecular development in the ovary of the blotched snakehead. This study showed that dietary supplementation with 3.0% ARA enhanced the transcriptional level of *tgfb1* in the ovary, which in turn activated the downstream factors *smad2* and *smad3*, but not *smad4*, as well as increased the protein levels of TGF-β1, Smad2, Smad3, and Smad4. This suggests that the promotion of steroid hormone synthesis by ARA and the ultimate maturation of the ovary might be associated with the activation of the TGF-β1/Smads signaling pathway. The TGF-β1/Smads signaling pathway plays a crucial role in ovarian follicular proliferation, steroidogenesis, cell differentiation and luteal formation, and also regulates the secretion of gonadotropins in the pituitary gland [42]. The downstream effector factors of TGF-β1, namely SMAD2 and SMAD3, are crucial for follicular growth and play unique roles in granulosa cell development. It has been found in zebrafish that TGF-β1 plays a significant role in the transformation of primary growth oocytes into previtellogenic oocytes [21]. In addition, the results of this study further indicated that dietary ARA could up-regulate the transcription levels of *cyp19a1*, *er*, and *pr*, as well as activate the expression of *foxo1* and *foxo3* genes. This finding further confirms the existence of a dose-dependent relationship between ARA and the regulation of ovarian steroid hormone synthesis. In female Atlantic cod (*Gadus morhua* L.), plasma concentrations of E2 and T, as well as transcript levels of the key genes P450 aromatase (*cyp19a1a*) and 20β-hydroxysteroid dehydrogenase (*20β-hsd*), were affected by dietary ARA (0.5–4.0% of total fatty acids) [43]. Numerous studies have confirmed that both *foxo1* and *foxo3* exhibit high expression levels in the granulosa cells of the teleost ovary, participate in the regulation of lipid and steroid biosynthesis, and play a crucial role in ovarian maturation [44,45]. Given that the TGF-β/Smad signaling pathway is evolutionarily conserved and has been well characterized across multiple vertebrate taxa, including teleosts, we hypothesize that dietary ARA promotes ovarian follicle development and oocyte maturation in blotched snakehead via activation of the ovarian TGF-β/Smad signaling cascade.

Metabolomics studies are crucial for delineating a more comprehensive portrait of the alterations in metabolites within the ovaries and larvae of the broodstock under ARA feeding conditions. In the current study, the differential metabolites in both parental and offspring larvae were significantly enriched in the ARA metabolism and VEGF signaling pathways, suggesting that dietary ARA supplementation can regulate ovarian development and offspring health by influencing these signaling pathways. It has been confirmed that ARA and its metabolites act as second messengers in the regulation of the cardiovascular, immune, and neuroendocrine systems [46]. The differential metabolites in the ovaries and larvae were screened and subjected to clustering analysis. The metabolites of ARA, including prostaglandin I2, prostaglandin F1, 12-keto leukotriene B4, and prostaglandin J2, showed a substantial increase in the ARA3.0 group. Research has demonstrated that ARA-derived eicosanoids serve as mediators in the reproductive regulatory effects of ARA. In a study on Eurasian perch, it was discovered that PGE2 and PGF2α may potentially impact the final maturation of oocytes [5]. Moreover, the cAMP produced in response to PGE2 appears to be regulated via an intracellular pathway involving a PGE2-like receptor [5]. Our results found that the abundances of differential metabolites, including leukotriene D4, 6-keto prostaglandin E2, 6-keto prostaglandin F1α, prostaglandin I2, 12-keto leukotriene B4, prostaglandin J2, and lysophosphatidylcholine, were significantly increased in the larvae of the ARA3.0 group.

Lysophosphatidylcholine is the primary glycerophospholipid component of fish egg yolk, and it participates in membrane remodeling during oocyte growth, while providing lipid substrates for early embryonic development and larval development via maternal nutrient transfer [47]. Although KEGG enrichment analysis indicated alterations in VEGF signaling and glycolysis/gluconeogenesis pathways, no highly abundant core signature metabolites in our dataset were found to be directly mapped to these two pathways. Enrichment of the VEGF signaling pathway may indirectly reflect alterations in ovarian follicular angiogenesis associated with improved follicle quality, as VEGF-dependent vascular remodeling supports follicular proliferation and maturation in teleost ovaries [21]. Enrichment of the glycolysis/gluconeogenesis pathway indicates potential shifts in the energy metabolic profile of fast-growing follicular cells. However, causal associations between VEGF signaling and glycolysis-associated responses remain unconfirmed in the present study. Lipid-derived eicosanoids and lysophosphatidylcholines are the most plausible candidate metabolites that link dietary ARA supplementation to the improved reproductive performance of blotched snakehead. These findings suggest that, in addition to directly regulating reproduction itself, dietary ARA exerts a more substantial effect on the offspring of broodstock.

## 4. Materials and Methods

### 4.1. Experimental Diets

Three experimental diets were formulated to have comparable crude protein (43.1%) and lipid (10.5%) levels. The main protein sources included fishmeal, corn gluten meal, soybean meal, and wheat flour. Soybean oil (4%) was included in all feed formulations, with ARA-enriched oil replaced with tristearin to maintain isocaloric substitution. The control group received no ARA-enriched oil, while the experimental groups were supplemented with 1.5% and 3.0% ARA-enriched oil, designated as C, ARA1.5, and ARA3.0, respectively. The ingredient compositions and fatty acid profiles of the diets are presented in Tables S1 and S2, respectively. Feed ingredients were finely ground, thoroughly mixed for 15 min, and pelleted into 4.0 mm diameter granules using an extrusion system. The diets were dried at 70 °C for approximately 45 min in a forced-air oven, then sealed and stored under light-protected conditions.

The proximate composition of the experimental diets was quantified via standard protocols [48]. Specifically, crude protein content was calculated as nitrogen (N) multiplied by 6.25 and was analyzed using a Kjeltec System (FOSS Tecator; Haganäs, Sweden), following AOAC method 928.08. Crude lipid levels were determined by ether extraction method with a Soxtec System HT (Tecator, Haganäs, Sweden) in accordance with AOAC method 991.36. Crude ash content was measured by incineration at 550 °C for 12 h in a muffle furnace (Thermolyne, Waltham, MA, USA), adhering to AOAC method 920.153. Gross energy was determined using an Automated Oxygen Bomb Calorimeter (ATC 300A, Zeal Instruments, Zhejiang, China).

### 4.2. Feeding Trial Conditions

Blotched snakeheads were obtained from Foshan Zhenliang Aquaculture Co., Ltd. (Foshan, China), and shared the same genetic background and nutritional history. All fish were kept in pond cages for one month during the overwintering period. During this time, they were provided with a commercial diet once a day. After overwintering, a total of 360 females with an average initial body weight of (852.78 ± 30.51) g were randomly distributed into 9 cages (1.5 m × 1.5 m × 1.5 m), with 40 fish per cage and 3 replicates per treatment corresponding to three experimental diets. Throughout the 12-week experimental period, fish were fed twice daily at 7:00 and 17:00 to apparent satiation. Water temperature was maintained between 25 and 29 °C, with dissolved oxygen levels consistently kept above 5 mg/L. The pH remained within the range of 6.6 to 7.9.

### 4.3. Sample Collection

At the conclusion of the feeding trial, three female fish were randomly chosen from each cage and anesthetized with 80 mg/L MS-222. Body length and body weight were precisely recorded, after which blood samples were collected from the caudal vein using heparinized syringes. Blood was centrifuged at 3000× *g* for 5 min at 4 °C, and the resulting serum was separated and stored at −80 °C for subsequent hormone analysis. Following blood collection, fish were dissected to remove visceral organs; the liver and ovary were carefully excised and weighed individually to calculate the hepatosomatic index (HSI) and gonadosomatic index (GSI), respectively. Small aliquots of ovarian and muscle tissue were sampled and preserved at −80 °C for further molecular and biochemical analyses. Additionally, a portion of ovarian tissue was preserved in 4% paraformaldehyde for the immunohistochemical analysis.

### 4.4. Spawning, Egg and Larval Collection

Five male blotched snakeheads from the same family line were used in the artificial insemination experiment and reared in floating net cages on a commercial diet during the spawning season to ensure breeding readiness. Nine female blotched snakeheads from each group were randomly selected for reproductive performance evaluation. Ovulation was induced via intramuscular injection of human chorionic gonadotropin (HCG) and luteinizing hormone-releasing hormone A2 (LHRH-A2; Ningbo No. 2 Hormone Factory, Ningbo, China). Each female received two intra-peritoneal injections. The first injection contained 400 IU HCG plus 6 μg/kg body weight LHRH-A2, and the second injection contained 1000 IU HCG plus 12 μg/kg body weight LHRH-A2; all dosages were calculated based on individual fish body weight. Males were administered the second injection simultaneously with females, using half the dose. When the females reached effect time, five males were anesthetized using MS-222 (100 mg/L, Sigma, New York, NY, USA). Subsequently, the testis was dissected, minced, and mixed with a small volume of preservation solution, followed by filtration to obtain sperm suspension. Eggs were collected by gently pressing the abdomen of the female fish, after which approximately 2 mL of the sperm suspension was added, and the gametes were mixed thoroughly for 20 s to achieve artificial insemination.

Fertilized eggs collected from each female were placed into a rectangular incubation tank (60 cm × 40 cm × 40 cm) and incubated under stable thermal conditions at 28 °C. To determine the fertilization rate, approximately 300 eggs were randomly selected from each female fish for static water incubation. Following the observation of gastrulation, the total number of eggs and the number of successfully fertilized eggs were counted. The malformation rate was defined as the percentage of larvae with abnormal body curvature [49]. The same batch of fertilized eggs was further cultivated until the hatching period, and the number of larvae was recorded to calculate the hatching rate. All measurements were performed in triplicate. From each group, ninety larvae were randomly selected at 3 and 7 days post-hatching to measure body length and body weight. Reproductive performance was calculated according to the following formulae:

Fertilization rate (%) = 100 × total fertilized eggs/total number of eggs;

Hatching rate (%) = 100 × total number of larvae/total number of fertilized eggs;

Malformation rate (%) = 100 × total malformed larvae/total number of larvae;

Absolute fecundity = total eggs produced per female;

Relative fecundity = total egg production per female/mean weight of female.

### 4.5. Plasma Hormone Levels

The plasma concentrations of 11-keto-testosterone (11-KT, cat. H088-1-1), 17β-estradiol (E2, cat. H102-1-1), luteinizing hormone (LH, cat. H206-1-2), prostaglandin E2 (PGE2, cat. H099-1-2), and prostaglandin F2α (PGF2α, cat. H442-1) were quantified using commercial ELISA kits (Nanjing Jiancheng Bioengineering Institute, Nanjing, China). According to the manufacturer’s specifications, the minimum detectable sensitivities for 11-KT, E2, LH, PGE2 and PGF2α were 0.1 ng/mL, 1.0 pg/mL, 0.1 mIU/mL, 1.0 pg/mL and 2.5 pg/mL, respectively. The intra-assay coefficient of variation (CV) < 10% and inter-assay CV < 12%. Microplate absorbance was read at 450 nm using a microplate reader. Standard curves were generated on each plate, and final hormone concentrations were calculated after correction for sample dilution factors and initial sample volumes.

### 4.6. Ovarian Immunohistochemistry

Immunohistochemistry was performed on ovarian tissues to evaluate the protein levels of estrogen receptor (ER) and aromatase cytochrome P450 (CYP19A) receptor. All antibodies, reagents and experimental protocols used in the present study were consistent with those reported in reference [27]. The simplified protocol is as follows: After deparaffinization, rehydration and antigen retrieval, sections were blocked with 3% H_2_O_2_ and 5% BSA, then incubated with primary antibodies (1:200, Proteintech, Rosemont, IL, USA) overnight at 4 °C and HRP-conjugated secondary antibodies (1:500, Yuanye Bio-Technology, Shanghai, China) for 1 h at room temperature. Staining was visualized with DAB, followed by counterstaining with Mayer hematoxylin, dehydrated, cleared and mounted. For the negative control group, the primary antibodies were omitted and replaced with antibody diluent.

### 4.7. Fatty Acid Analysis

Total lipids were extracted from the feed and tissue samples using 5 mL chloroform/methanol (2:1, vol/vol), followed by transesterification with methanolic sulfuric acid to yield fatty acid methyl esters (FAMEs). The FAMEs were transferred to a new centrifuge tube, and 1 mL of 0.4 mol/L KOH-methanol solution was added. The mixture was vortexed for 3 min and then left to stand for 30 min. After the addition of ultrapure water, the upper aqueous layer was collected for subsequent processing. The harvested upper layer (200 μL) was mixed with 800 μL of hexane, filtered through a 0.22-μm syringe filter prior to fatty acid profiling via gas chromatography (GC-2010, Shimadzu Corporation, Kyoto, Japan). Calibration curves were generated through serial dilution of a FAME standard mixture. Fatty acid identification and quantification were performed by matching retention times and integrating peak areas, calibrated against certified FAME reference standards (Sigma-Aldrich, St. Louis, MO, USA).

### 4.8. Western Blot

Total proteins were extracted from ovarian tissues using RIPA lysis buffer (BL504A, Biosharp, Beijing, China) supplemented with Protease Inhibitor Cocktail (04693159001, ROCHE, Basel, Switzerland), phenylmethanesulfonyl fluoride (PMSF, G2008-1ML, Servicebio, Wuhan, China), and phosphatase inhibitors (G2007-1ML, Servicebio). Tissues were rinsed with pre-chilled PBS, minced, homogenized in 10× lysis buffer on ice, and incubated for 30 min with intermittent pipetting to ensure complete lysis. After centrifugation at 13,000× *g* for 5 min at 4 °C, supernatants were collected and quantified via the bicinchoninic acid protein assay kit (G2026-1000T, Servicebio). Equal quantities (40 μg) of denatured proteins, which were mixed with 5× protein loading buffer (BL502B, Biosharp), were loaded onto sodium dodecyl sulfate–polyacrylamide gel electrophoresis (SDS-PAGE) gels. SDS-PAGE was run at 50 V for the stacking gel and 120 V for the separating gel using electrophoresis buffer (Ba1013, Baiqiandu, Wuhan, China). Separated proteins were electrotransferred onto methanol-activated polyvinylidene fluoride membranes at 100 V with transfer buffer (Ba1015, Baiqiandu). Membranes were blocked with 5% skimmed milk in Tris-buffered saline containing 0.1% Tween-20, incubated with primary antibodies overnight at 4 °C, followed by incubation with secondary antibodies for 30 min at room temperature, and then thoroughly washed with TBST. The primary antibodies GAPDH (ab181602), TGF-β1 (ab215715), SMAD2 (ab40855), SMAD3 (ab40854), and SMAD4 (ab40853) were purchased from Abcam. These commercial antibodies were originally raised against mammalian antigens; however, prior to performing the Western blot experiments, an in silico sequence alignment was performed to compare the immunogen sequences and a high amino acid sequence identity was observed in the conserved functional domains of TGF-β1, Smad2, Smad3, Smad4 and GAPDH between blotched snakehead and the corresponding mammalian immunogen regions. Chemiluminescence detection was performed using an enhanced chemiluminescence reagent (MA0186, Meilunbio, Dalian, China), and images were acquired using a chemiluminescence imaging system. The optical density of target bands was quantified using Image J 1.52u8 software.

### 4.9. Real-Time Quantitative PCR

Total RNA was extracted from ovarian samples using TRIzol reagent, and its integrity was examined by 1% agarose gel electrophoresis. qPCR was performed on a StepOnePlus Real-Time PCR System (Applied Biosystems, Waltham, MA, USA) with SYBR Green Master Mix (Toyobo Co., Ltd., Osaka, Japan) in 20 μL reactions, which included 1.0 μL of each primer, 10 μL of 2× SYBR Green I Master mix, 2 μL of ten-fold-diluted cDNA template, and 6 μL of diethyl pyrocarbonate-treated water. The amplification process was performed at 95 °C for 2 min, followed by 40 cycles of 95 °C for 10 s, 60 °C for 10 s, and 72 °C for 20 s. β-actin was selected as the internal reference gene based on pre-experimental validation. The relative mRNA expression was calculated by the 2^−ΔΔCT^ method. Primer sequences are shown in Table S3.

### 4.10. Metabolomic Analysis

Samples of ovaries and 3DPH larvae from the control and ARA3.0 groups were taken for untargeted metabolomics analysis. The collected samples were rapidly frozen in liquid nitrogen and then ground into a fine, uniform powder using a tissue homogenizer (60 Hz, 2 min). Metabolite extraction was conducted by mixing 50 mg of the powdered sample with 1 mL of ice-cold methanol–water solution (4:1, *v*/*v*) spiked with an internal standard (L-2-chlorophenylalanine, 1 μg/mL). The mixture was vigorously vortexed for 30 s and subjected to ultrasonic extraction on ice for 30 min. After centrifugation (13,000× *g*, 15 min, 4 °C), the supernatant was filtered through a 0.22 μm organic-phase filter and transferred to LC-MS vials. Untargeted metabolomic profiling was performed using an ultra-high-performance liquid chromatography (UHPLC) system combined with a quadrupole-time-of-flight mass spectrometer (Q-TOF MS). Mass spectrometry was performed in both positive and negative electrospray ionization (ESI) modes using the following parameters: capillary voltage (3.5 kV in positive mode, −3.0 kV in negative mode), ion source temperature (120 °C), desolvation temperature (350 °C), desolvation gas flow rate (800 L/h), and cone gas flow rate (50 L/h). Full-scan spectra (*m*/*z* 50–1000) were acquired, and MS/MS fragmentation was performed using collision energies ranging from 10 to 40 eV. Metabolite identification was achieved by matching accurate mass (mass deviation < 10 ppm), MS/MS spectra, and retention time with entries in public databases such as HMDB, METLIN, and KEGG. Orthogonal Projections to Latent Structures Discriminant Analysis (OPLS-DA) was employed to validate the robustness of the model. Differential metabolites were screened using a combined criterion integrating fold change (FC), *p* value, and VIP values derived from the OPLS-DA model, with the thresholds set as FC > 2, *p* value < 0.05, and VIP > 1. The significance of KEGG pathway enrichment for the identified differential metabolites was assessed using the hypergeometric distribution test.

### 4.11. Statistical Analysis

The data were presented as means and SEM with the number of biological replicates indicated. Normality and homogeneity of variance were assessed using the Shapiro–Wilk test and Levene’s test, respectively. Statistical analysis was conducted using one-way ANOVA, followed by Duncan’s test to evaluate significant differences among treatment groups. All statistical analyses were carried out using IBM SPSS Statistics 23 software (SPSS, Chicago, IL, USA). A statistically significant difference was considered at a threshold of *p* < 0.05.

## 5. Conclusions

In conclusion, dietary ARA supplementation at 3.0% of the diet improved the reproductive performance in female blotched snakehead, as evidenced by increased fertilization and hatching rates, decreased larval malformation rate, and enhanced post-hatching larval growth. Dietary ARA supplementation elevated the concentrations of plasma sex steroid hormones and prostaglandins, and promoted the deposition of HUFA in ovarian tissue. At the molecular level, ARA up-regulated the expression of ovarian *tgfb1*, *smad2*, *smad3*, *cyp19a1*, *er* and steroid synthesis-related genes and increased the protein abundance of TGF-β1, Smad2, Smad3 and Smad4. These findings support the conclusion that the TGF-β/Smad signaling pathway mediates ARA-promoted ovarian maturation. Furthermore, untargeted metabolomics analysis of ovarian and larval tissues revealed alterations in ARA-derived eicosanoids and lipid metabolites, indicating that ARA exerts maternal nutritional programming effects on offspring larvae. Collectively, these results demonstrate that appropriate dietary ARA can improve the reproductive output of broodstock blotched snakehead and the quality of their offspring larvae.

## Figures and Tables

**Figure 1:**
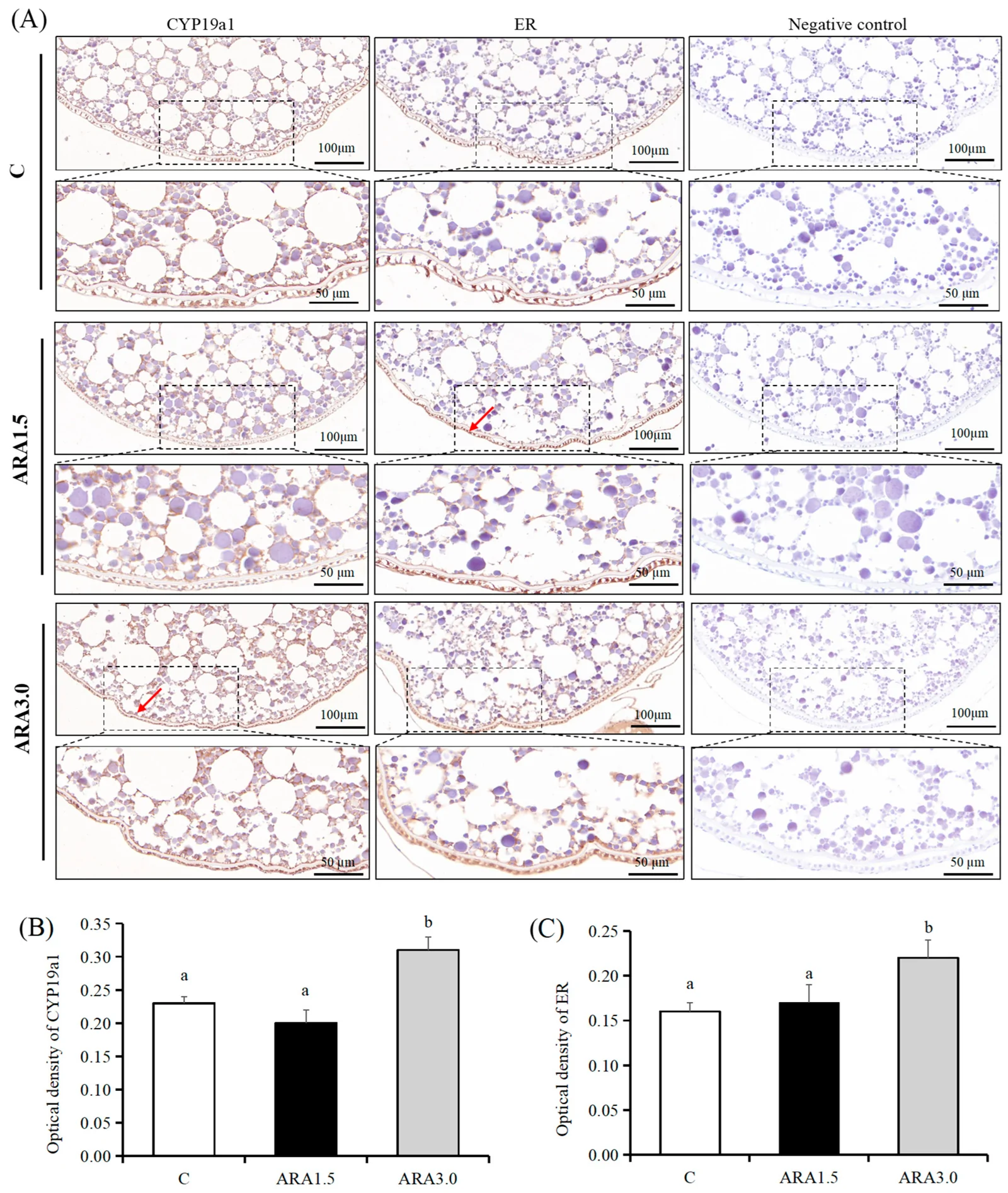
Immunohistochemical analysis of CYP19A1 and ER in the ovaries of broodstock blotched snakehead fed diets with different ARA levels. (**A**) Immunohistochemical staining. The positive antigen was dyed brown with 3′,3′-diaminobenzidine (DAB) (arrows). Negative controls: no positive signals were observed in the negative control (cells are blue). (**B**,**C**) The optical density of CYP19A1 and ER in the ovary. Different lowercase letters above columns represent significant differences among treatments at *p* < 0.05 (n = 6). CYP19A1 = cytochrome P450 family 19 subfamily a member 1; ER = estrogen receptor.

**Figure 2:**
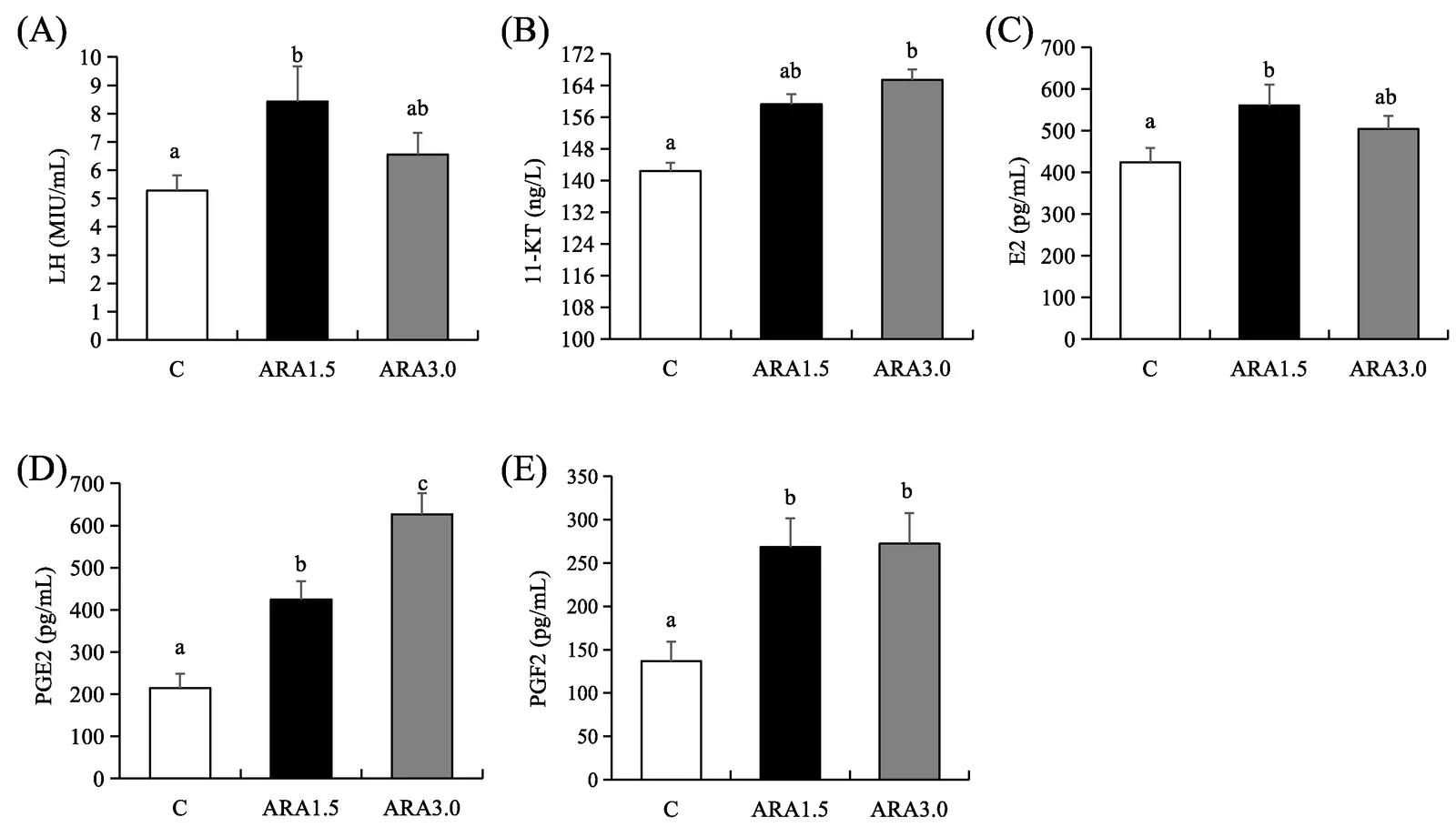
Effects of dietary ARA levels on plasma sex hormone levels of blotched snakehead broodstock. a–c: The bar graph with different lowercase letters indicates that the difference is significant (*p* < 0.05). Values are presented as mean ± SEM (*n* = 6). 11KT = 11-keto testosterone; E2 = 17β-estradiol; PGE2 = Prostaglandin E2; PGF2 = Prostaglandin F2α; LH = Luteinizing hormone. (**A**) Luteinizing hormone; (**B**) 11-keto testosterone; (**C**) 17β-estradiol; (**D**) Prostaglandin E2; (**E**) PGF2 = Prostaglandin F2α.

**Figure 3:**
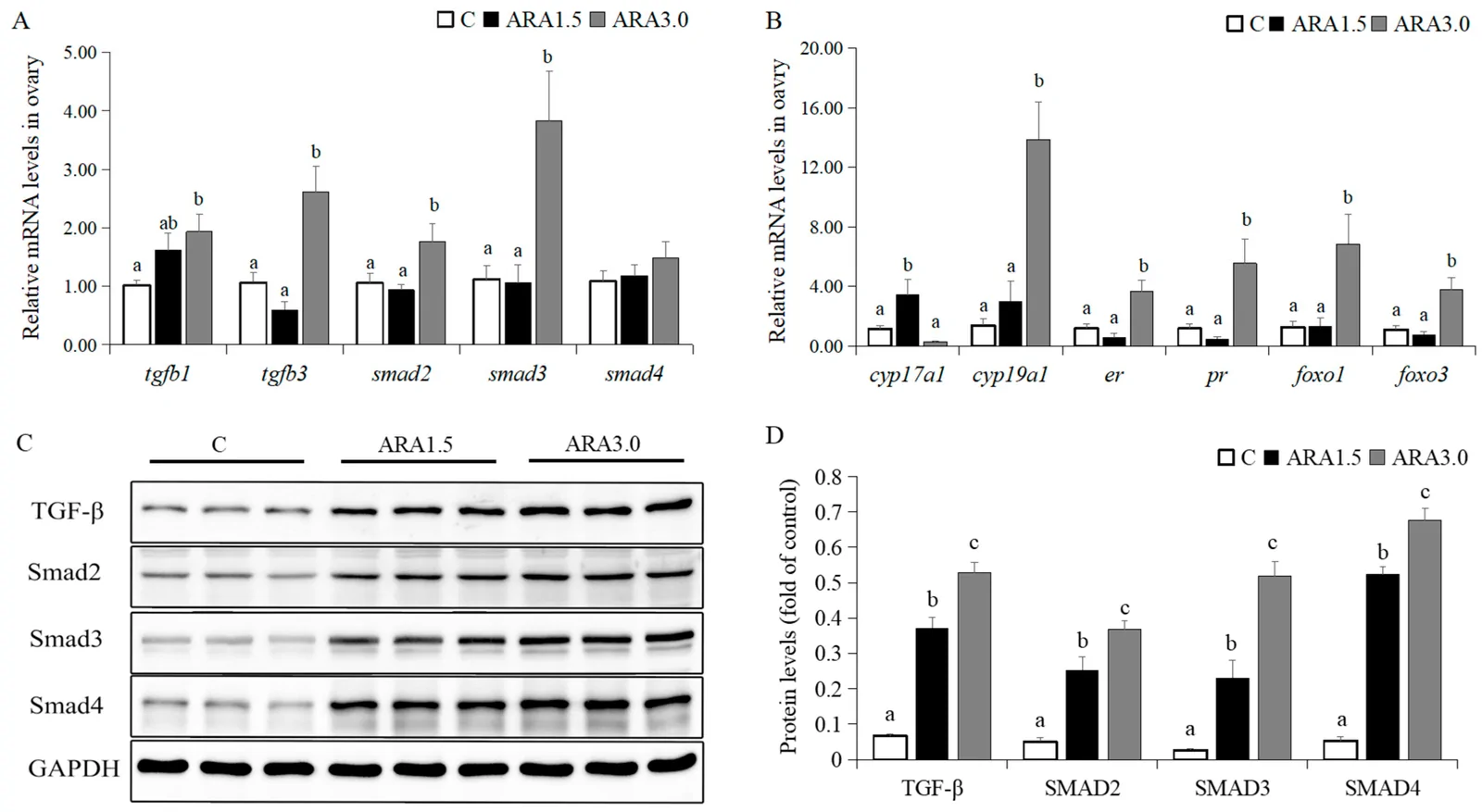
Effects of dietary ARA on TGF-β/Smad signaling pathway and steroid hormone synthesis in broodstock blotched snakehead. (**A**) Expression of genes associated with the TGF-β/Smad signaling pathway in ovary (*n* = 6). (**B**) Expression of genes associated with steroid hormone synthesis in ovary (*n* = 6). (**C**,**D**) Expression of TGF-β, Smad2, Smad3 and Smad4 was detected by Western blotting and corresponding quantification (*n* = 3). Values are the mean ± SEM. Different letters denote significant differences (*p* < 0.05).

**Figure 4:**
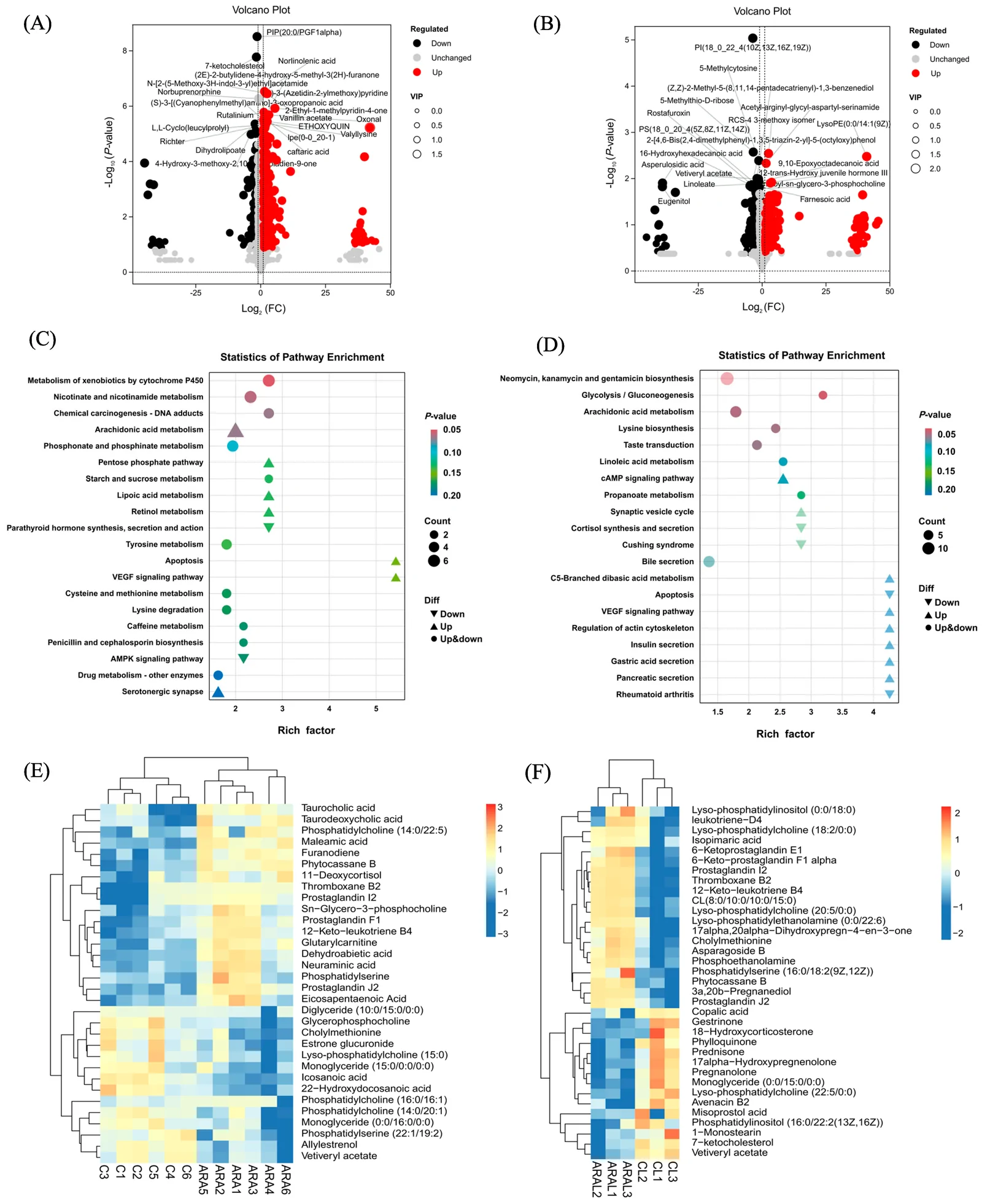
Statistical comparison of metabolites and analysis of differential metabolites and key metabolic pathways. (**A**,**B**) Volcano map of DEMs in the ovary and 3DPH larvae from comparisons between C vs. ARA3.0. The vertical and horizontal axes display the expression levels of the metabolites in the two groups using -log_10_ (*p*-value) and log_2_ (FC), respectively. Each point in the graph represents a specific metabolite whose size corresponds to the VIP value. Red dots: significantly up-regulated metabolites (VIP > 1 and *p* < 0.05). Dark dots: significantly down-regulated metabolites (VIP > 1 and *p* < 0.05). Gray dots: no significant difference (VIP < 1 and *p* > 0.05). (**C**,**D**) Kyoto Encyclopedia of Genes and Genomes (KEGG) enrichment analysis of the DEMs in the ovary and 3DPH larvae from comparisons between C vs. ARA3.0. The figure shows the pathway list of the top 20 smallest *p*-values. FC = fold change; VIP = variable important in projection. (**E**,**F**) A heatmap of the lipids and lipid-like molecules as differentially expressed metabolites (DEMs) in the ovaries and 3DPH larvae from comparisons between C and ARA3.0. The colors indicate the expression levels of the metabolites in the sample group.

**Table 1:** Reproductive performance of blotched snakehead broodstock fed different ARA diets for 12 weeks.

Item	C	ARA1.5	ARA3.0
IBW, g	849.61 ± 41.75	855.61 ± 43.55	850.61 ± 38.68
FBW, g	946.86 ± 57.53	997.82 ± 53.08	971.08 ± 51.13
HSI, %	1.41 ± 0.12 ^a^	1.88 ± 0.11 ^ab^	2.02 ± 0.22 ^b^
GSI, %	11.14 ± 0.55	10.77 ± 0.46	9.99 ± 0.54
CF, g/cm^3^	2.12 ± 0.15	2.12 ± 0.12	2.12 ± 0.11
FI, %/(fish·d)	0.91 ± 0.12	0.95 ± 0.09	0.89 ± 0.08
Absolute fecundity	43,475.58 ± 2000.42	48,211.17 ± 2360.55	43,685.12 ± 1803.63
Relative fecundity	46.32 ± 2.07	48.55 ± 1.87	45.98 ± 3.85
Fertilization rate, %	73.86 ± 4.62 ^a^	86.43 ± 4.35 ^b^	91.91 ± 2.66 ^b^
Hatching rate, %	20.74 ± 2.79 ^a^	41.89 ± 1.99 ^b^	52.38 ± 2.40 ^c^
Malformation rate, %	13.35 ± 1.44 ^b^	11.23 ± 1.62 ^b^	6.99 ± 1.06 ^a^
Egg diameter, mm	1.57 ± 0.09 ^b^	1.50 ± 0.08 ^a^	1.47 ± 0.07 ^a^
Larvae body weight 3DPH, mg	2.84 ± 0.05	2.43 ± 0.03	3.45 ± 0.05
Larvae body length 3DPH, mm	5.90 ± 0.55 ^a^	6.91 ± 0.26 ^b^	6.75 ± 0.40 ^ab^
Larvae body weight 7DPH, mg	4.63 ± 0.07 ^a^	4.95 ± 0.06 ^b^	4.84 ± 0.06 ^b^
Larvae body length 7DPH, mm	7.34 ± 0.47 ^a^	7.60 ± 0.37 ^b^	7.50 ± 0.34 ^ab^

IBW = initial body weight; FBW = final body weight; HSI = hepatosomatic index; GSI = gonadsomatic index; FI = 100 × total feed intake/days/[(IBW + FBW)/2]; CF = 100 × (Body weight/Body length^3^). ^a,b,c^ Values in the same row with different letter superscripts are significantly different (*p* < 0.05). Values are presented as mean ± SEM.

**Table 2:** Effect of dietary ARA levels on the ovarian and muscle fatty acid profile (g/kg, wet weight) of blotched snakehead broodstock.

Item	Ovaries			Muscle		
	C	ARA1.5	ARA3.0	C	ARA1.5	ARA3.0
C14:0	1.47 ± 0.22	2.08 ± 0.19	1.99 ± 0.27	0.53 ± 0.07	0.59 ± 0.08	0.83 ± 0.13
C16:0	11.43 ± 1.73	14.50 ± 1.08	12.63 ± 0.99	4.29 ± 0.31	4.78 ± 0.62	5.77 ± 0.38
C18:0	2.93 ± 0.56	3.61 ± 0.09	3.07 ± 0.19	1.02 ± 0.06	1.14 ± 0.17	1.27 ± 0.05
Total SFA	15.83 ± 2.51	20.19 ± 1.36	17.70 ± 1.43	5.84 ± 0.38	6.51 ± 0.87	7.18 ± 0.38
C16:1n-9	9.15 ± 0.74	10.11 ± 0.35	9.59 ± 1.25	0.841 ± 0.12	0.90 ± 0.13	1.08 ± 0.14
C18:1n-9	65.43 ± 5.11	63.03 ± 1.21	55.13 ± 2.00	4.53 ± 0.35	5.14 ± 0.84	5.70 ± 0.28
C20:1n-9	5.72 ± 0.40	6.13 ± 0.18	5.39 ± 0.16	0.46 ± 0.05	0.51 ± 0.05	0.60 ± 0.02
Total MUFA	80.30 ± 6.07	79.27 ± 0.98	70.12 ± 3.20	6.04 ± 0.52	6.77 ± 1.02	7.59 ± 0.15
C18:2n-6	45.87 ± 1.77 ^b^	38.40 ± 2.29 ^ab^	34.57 ± 2.38 ^a^	2.26 ± 0.35	2.72 ± 0.65	2.84 ± 0.35
C18:3n-3	3.73 ± 0.22	3.42 ± 0.16	3.24 ± 0.10	0.20 ± 0.01	0.25 ± 0.08	0.26 ± 0.02
C20:2n-6	1.66 ± 0.01 ^b^	1.53 ± 0.09 ^ab^	1.29 ± 0.13 ^a^	0.12 ± 0.02	0.14 ± 0.02	0.14 ± 0.02
C20:4n-6 (ARA)	1.25 ± 0.10 ^a^	3.18 ± 0.19 ^b^	4.88 ± 0.73 ^c^	0.17 ± 0.01 ^A^	0.22 ± 0.04 ^A^	0.35 ± 0.05 ^B^
C20:5n-3 (EPA)	2.61 ± 0.15 ^a^	4.48 ± 0.18 ^b^	6.22 ± 0.85 ^b^	0.42 ± 0.03 ^A^	0.49 ± 0.07 ^A^	0.69 ± 0.04 ^B^
C22:6n-3 (DHA)	14.13 ± 1.16 ^a^	18.33 ± 0.41 ^ab^	21.30 ± 2.08 ^b^	1.96 ± 0.14 ^A^	2.13 ± 0.32 ^AB^	2.79 ± 0.21 ^B^
Total n-3 PUFA	20.47 ± 1.30 ^a^	26.23 ± 0.33 ^ab^	30.76 ± 2.99 ^b^	2.59 ± 0.18 ^A^	2.88 ± 0.40 ^AB^	3.745 ± 0.20 ^B^
Total n-6 PUFA	48.90 ± 1.78 ^b^	42.94 ± 2.33 ^ab^	40.89 ± 1.86 ^a^	2.55 ± 0.37	3.08 ± 0.67	3.34 ± 0.39
Total HUFA	71.03 ± 2.27	70.67 ± 2.17	72.94 ± 1.07	5.14 ± 0.53	5.95 ± 0.88	7.08 ± 0.54

SFA = saturated fatty acid; MUFA = monounsaturated fatty acid; ARA = arachidonic acid; EPA = eicosapentaenoic acid; DHA = docosahexaenoic acid; PUFA = polyunsaturated fatty acid; HUFA = highly unsaturated fatty acid. Within a row, means without a common superscript letter differ at *p* < 0.05, *n* = 6.

## Data Availability

The original contributions presented in this study are included in the article/Supplementary Materials. Further inquiries can be directed to the corresponding author.

## Supplementary Materials

The following supporting information can be downloaded at: https://www.mdpi.com/article/10.3390/ijms27198540/s1.
