# Supplementary material for: Arachidonic Acid Enhances Reproductive Performance and Oocyte Quality of Female Blotched Snakehead (Channa maculata) via Activation of the TGF-β/Smad Signaling Pathway
Source: Int J Mol Sci. 2026 Sep 24;27(19):8540. doi: 10.3390/ijms27198540 (PMC13665339; doi:10.3390/ijms27198540)
Supplement: Supplementary file 1 [file ijms-27-08540-s001.zip › Supplementary Figure S1 Orthogonal partial least squares-discriminant analysis (OPLS-DA) analyses.pdf]

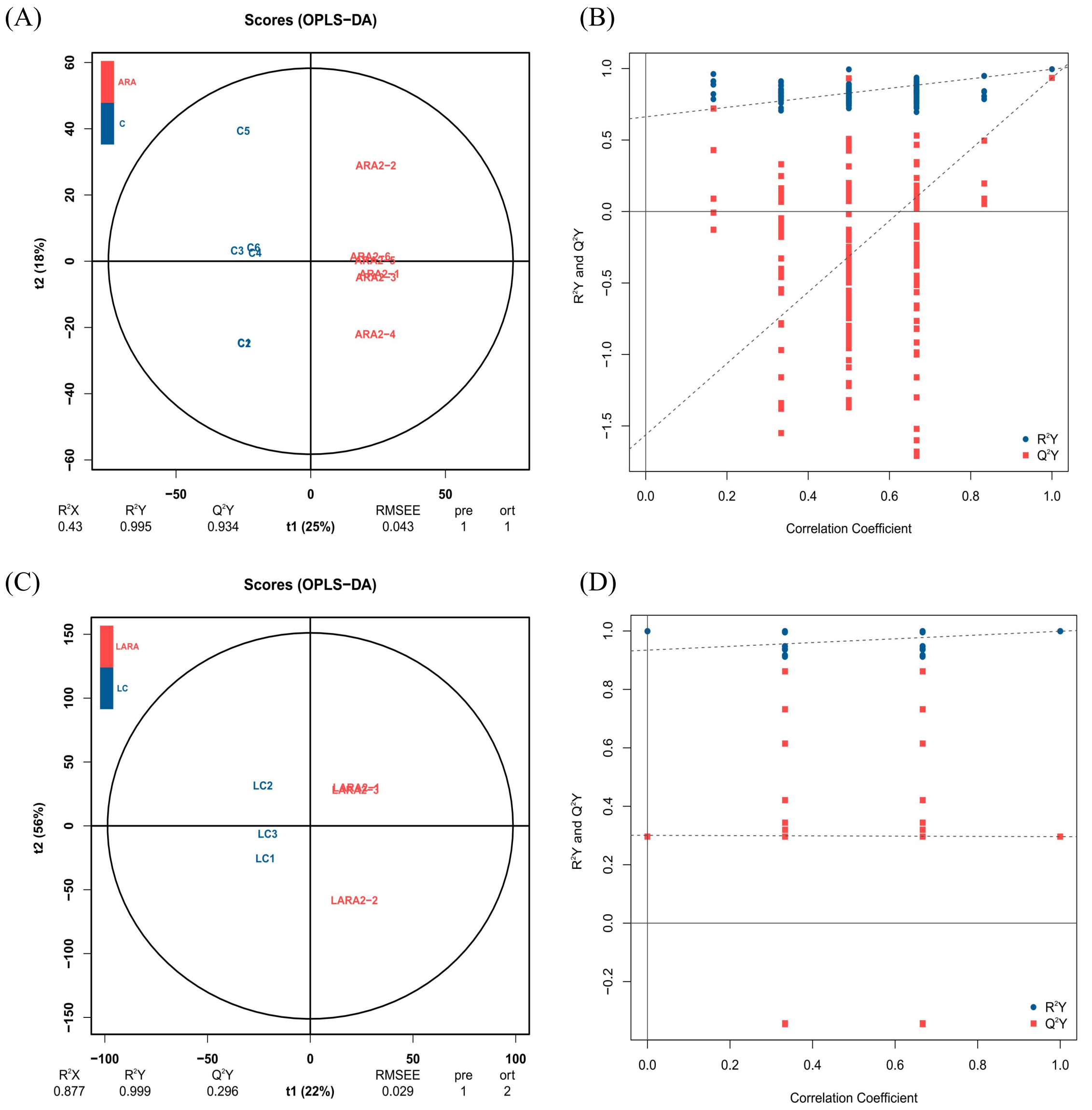

Figure S1. Orthogonal partial least squares-discriminant analysis (OPLS-DA) analyses and permutation tests for samples in different comparison groups. (A and B) OPLS-DA score plots and permutation test between C and ARA3.0 in the ovary; (C and D) OPLS-DA score plots and permutation test between C and ARA3.0 in the 3DPH larvae.
