# Supplementary material for: Arachidonic Acid Enhances Reproductive Performance and Oocyte Quality of Female Blotched Snakehead (Channa maculata) via Activation of the TGF-β/Smad Signaling Pathway
Source: Int J Mol Sci. 2026 Sep 24;27(19):8540. doi: 10.3390/ijms27198540 (PMC13665339; doi:10.3390/ijms27198540)
Supplement: Supplementary file 1 [file ijms-27-08540-s001.zip › Supplementary Table S1 Feed formulation and proximate analysis of experimental diets (dry matter basis, %)..pdf]

Table S1. Feed formulation and proximate analysis of experimental diets (dry matter basis, %).

Ingredients	C	ARA1.5	ARA3.0
Fishmeal	37.0	37.0	37.0
Corn gluten meal	15.0	15.0	15.0
Soybean meal	16	16.0	16.0
Wheat flour	5.0	5.0	5.0
Cassava starch	12.2	12.2	12.2
ARA-enriched oil [1]	0	1.5	3.0
Tristearin [2]	3.0	1.5	0.0
Soybean oil	4.0	4.0	4.0
Vitamin premix [3]	0.5	0.5	0.5
Mineral premix [4]	5.0	5.0	5.0
Vitamin C	0.5	0.5	0.5
Squid paste	0.5	0.5	0.5
$Ca(H_2PO_4)_2$	1.0	1.0	1.0
Choline chloride	0.1	0.1	0.1
Ethoxyquinoline	0.2	0.2	0.2
Total	100	100	100
Proximate composition			
Crude protein	43.15	43.24	43.46
Crude lipid	9.38	9.4	9.54
Moisture	5.98	5.91	5.78
Gross energy, KJ/g	20.46	20.64	20.59

[1] ARA-enriched oil: containing 40% ARA (of total fatty acids) in the form of ARA-methyl ester. Xi 'an Mixianer Biotechnology Co., Ltd. Shaanxi, China

[2] Tristearin: Jiangsu Maoheng Chemical Co., Ltd. Jiangsu, China.

[3] Mineral premix (mg/kg diet): NaCl, 500.0; MgSO4·7H2O, 8155.6; NaH2PO4·2H2O, 12500.0; KH2PO4, 16000.0; Ca(H2PO4)2H2O, 7650.6; FeSO4·7H2O, 2286.2; C6H10CaO6·5H2O, 1750.0; ZnSO4·7H2O, 178.0; MnSO4·H2O, 61.4; CuSO4·5H2O, 15.5; CoSO4·7H2O, 0.91; KI, 1.5; Na2SeO3, 0.60; and Corn starch, 899.7.

[4] Vitamin premix (mg/ kg diet): Vitamin B1, 20; Vitamin B2, 20; Vitamin B6, 20; Vitamin B12, 0.02; folic acid, 5; calcium pantothenate, 50; inositol, 100; niacin, 100; biotin, 0.1; cellulose, 3522; Vitamin A, 11; Vitamin D, 2; Vitamin E, 100; and Vitamin K, 10
