# Supplementary material for: Arachidonic Acid Enhances Reproductive Performance and Oocyte Quality of Female Blotched Snakehead (Channa maculata) via Activation of the TGF-β/Smad Signaling Pathway
Source: Int J Mol Sci. 2026 Sep 24;27(19):8540. doi: 10.3390/ijms27198540 (PMC13665339; doi:10.3390/ijms27198540)
Supplement: Supplementary file 1 [file ijms-27-08540-s001.zip › Supplementary Table S2 Main fatty acid composition (g kg-1) of experimental diets..pdf]

Table S2. Main fatty acid composition (g kg^{-1} diet) of experimental diets.

g kg^{-1}	C	ARA1.5	ARA3.0
C14:0	1.25	1.26	1.24
C16:0	7.12	7.24	7.35
C18:0	1.69	1.64	1.68
Total SFA	10.06	10.14	10.27
C16:1n-7	2.63	2.72	2.74
C18:1n-9	8.57	8.61	9.19
C20:1n9	1.66	1.69	1.87
Total MUFA	12.86	13.02	13.80
C18:2n-6	10.63	12.44	13.91
C18:3n-6	0.18	0.94	1.48
C20:3n-6	0.32	0.39	0.33
C20:4n-6 (ARA)	0.84	7.76	13.35
Total n-6 PUFA	11.97	21.53	29.07
C18:3n-3	1.94	1.86	1.96
C20:3n-3	0.71	0.65	0.68
C20:5n-3 (EPA)	14.27	15.33	12.13
C22:6n-3 (DHA)	30.37	31.01	31.21
Total n-3 PUFA	47.29	48.85	45.98
Total HUFA	45.48	54.1	56.69
DHA/EPA	2.13	2.02	2.57
ARA/EPA	0.06	0.51	1.10

SFA = saturated fatty acid; MUFA = monounsaturated fatty acid; ARA = arachidonic acid; EPA = eicosapentaenoic acid; DHA = docosahexaenoic acid; PUFA = polyunsaturated fatty acid; HUFA = highly unsaturated fatty acid.
