# Supplementary material for: Arachidonic Acid Enhances Reproductive Performance and Oocyte Quality of Female Blotched Snakehead (Channa maculata) via Activation of the TGF-β/Smad Signaling Pathway
Source: Int J Mol Sci. 2026 Sep 24;27(19):8540. doi: 10.3390/ijms27198540 (PMC13665339; doi:10.3390/ijms27198540)
Supplement: Supplementary file 1 [file ijms-27-08540-s001.zip › Supplementary Table S3 The sequences information of primers involved in qRT-PCR.pdf]

Table S3. The sequences information of primers involved in qRT-PCR

Gene name	Forward sequences (5′–3′)	Reverse sequence (5′–3′)	Length (bp)	Temperature ℃
β-actin	AGCAAGCAGGAGTATGATGA	AGCAAGCAGGAGTATGATGA	283	60
cyp17a1	CAGTCACTTACCTCATCCATTATCC	TTTTTCCATTCCTTCTCGTCAT	283	58
cyp19a1	AATACCCCTCGTCGTTACTT	AAACCCTTATGGAGGCAAA	137	59
er	AGGTCCCGCGTCCTCTGTAT	CCTGTTGAACCCGTGAATGTG	146	58
tgfb3	TCCCTCGGACCAAATAAAATC	CGTTGTTCTCTGACATTCCGTAG	181	55
foxo1	CACAACTTATCACACCTC	TTAGTATTACCGTATCCA	234	60
pr	AACCCATCACCTTTTCCC	GACCTTCAACTGCTCTTT	142	60
foxo3	CAAATCCACAATCTCTAC	GTCACATTCCAACCTCTC	297	58
tgfb1	AAGGGTGTGAAATGCCCCAT	CACACTCGCCCGTATCTCAG	101	60
smad2	ATAGCCTACTATGAGCTAAA	ATATGTCTCCTTGTCATTTC	167	56
smad3	ATGTCAATTTTGCCTTTTAC	CTTCTTCACCAGACTTTTTA	120	59
smad4	TGGTAAAAGATGAGTATGAC	CTGATAGTAGAAAAAGCAGA	181	60

Abbreviations: cyp17a1, cytochrome P450 17a-hydroxylase/C17-20lyase; cyp19a1, cytochrome P450 aromatase; er, estrogen receptor; foxo3/1, forkhead box O3 /1; pr, progesterone receptor; tgfb1/3, transforming growth factor beta 1/3; smad2/3/4, SMAD family member 2/3/4.
